# IL-18 Induces Airway Hyperresponsiveness and Pulmonary Inflammation via CD4^+^ T Cell and IL-13

**DOI:** 10.1371/journal.pone.0054623

**Published:** 2013-01-29

**Authors:** Masanori Sawada, Tomotaka Kawayama, Haruki Imaoka, Yuki Sakazaki, Hanako Oda, Shin-ichi Takenaka, Yoichiro Kaku, Koichi Azuma, Morihiro Tajiri, Nobutaka Edakuni, Masaki Okamoto, Seiya Kato, Tomoaki Hoshino

**Affiliations:** 1 Division of Respirology, Neurology and Rheumatology, Department of Medicine 1, Kurume University School of Medicine, Fukuoka, Japan; 2 Division of Pathology and Cell Biology, Graduate School and Faculty of Medicine, University of the Ryukyus, Okinawa, Japan; University Hospital Freiburg, Germany

## Abstract

IL-18 plays a key role in the pathogenesis of pulmonary inflammatory diseases including pulmonary infection, pulmonary fibrosis, lung injury and chronic obstructive pulmonary disease (COPD). However, it is unknown whether IL-18 plays any role in the pathogenesis of asthma. We hypothesized that overexpression of mature IL-18 protein in the lungs may exacerbate disease activities of asthma. We established lung-specific IL-18 transgenic mice on a Balb/c genetic background. Female mice sensitized– and challenged– with antigen (ovalbumin) were used as a mouse asthma model. Pulmonary inflammation and emphysema were not observed in the lungs of naïve transgenic mice. However, airway hyperresponsiveness and airway inflammatory cells accompanied with CD4^+^ T cells, CD8^+^ T cells, eosinophils, neutrophils, and macrophages were significantly increased in ovalbumin-sensitized and challenged transgenic mice, as compared to wild type Balb/c mice. We also demonstrate that IL-18 induces IFN-γ, IL-13, and eotaxin in the lungs of ovalbumin-sensitized and challenged transgenic mice along with an increase in IL-13 producing CD4^+^ T cells. Treatment with anti-CD4 monoclonal antibody or deletion of the IL-13 gene improves ovalbumin-induced airway hyperresponsiveness and reduces airway inflammatory cells in transgenic mice. Overexpressing the IL-18 protein in the lungs induces type 1 and type 2 cytokines and airway inflammation, and results in increasing airway hyperresponsiveness via CD4^+^ T cells and IL-13 in asthma.

## Introduction

Asthma is a prevalent disease with annual worldwide deaths from asthma estimated at over 250,000 [1]. The inflammatory process in allergic asthma is initiated by T-helper 2 (Th2) CD4^+^ cells, which produce a repertoire of cytokines including IL-4, IL-5, IL-9 and IL-13. These cytokines play a critical role in IgE production, airway eosinophilia, and goblet cell hyperplasia [2]. Many previous studies have shown that activated CD4^+^ T cells, producing Th2 cytokines, were increased in the airways of mild asthmatics [3]. The Th2 cytokine, IL-13, may play a key role in increasing airway hyperresponsiveness (AHR), eosinophilic pulmonary inflammation, goblet cell metaplasia, and lung fibrosis [3]. In contrast, IFN-γ producing Th1 cells are thought to prevent asthma disease activity, but in some experimental models, Th1 cells cannot suppress Th2 cell-mediated AHR and pulmonary inflammation [3].

IL-18, a member of theInterleukin 1 (IL-1) family, is known as a pro-inflammatory cytokine [4], [5]. IL-18 is known to play an important role in Th1/Tc1 polarizationbut it also promotes Th2 cytokine (e.g. IL-4, IL-5, IL-9, and IL-13) production from T cells, NK cells, basophils, and mast cells. Thus, IL-18 can act as a co-factor for Th2 cell development and IgE production [6]–[9]. IL-18 also plays an important role in the pathogenesis of other inflammatory diseases such as atopic dermatitis, rheumatoid arthritis (RA), adult-onset Still’s disease, Sjögren’s syndrome, and inflammatory bowel diseases including Crohn’s disease [6] [10]–[11]. Furthermore, many lines of evidence suggest that IL-18 plays a key role in the pathogenesis of pulmonary inflammatory diseases including pulmonary infection, pulmonary fibrosis, lung injury and chronic obstructive pulmonary disease (COPD) [12]–[15]. However, the role of IL-18 is considered controversial in some experimental mouse asthma models [12] and it is still unknown whether overexpression of IL-18 in the lungs alters AHR and pulmonary inflammation in asthma. In this study, we examined whether overexpression of IL-18 protein in the lungs induces AHR and pulmonary inflammation in a mouse model of asthma.

## Materials and Methods

### Mice

We previously reported IL-18 transgenic (Tg) mice (C57BL/6N genetic background) in which the mature mouse IL-18 was overproduced in the lungs under the control of the human surfactant protein (SP) C promoter (hereafter IL-18 Tg mice) [16]. In this study, we establish Balb/c background IL-18 Tg mice by eight times backcrossing B6 IL-18 Tg mice and WT (wild-type) Balb/c mice. We also established Balb/c IL-13 deficient (−/−) mice by backcrossing 129 X B6 IL-13(−/−) mice [17] (kindly provided by Dr. Andrew N. McKenzie, Medical Research Council, UK) eight times with WT Balb/c mice. Furthermore, we established Balb/c IL-13 deficient (−/−) IL-18 Tg mice by backcrossing Balb/c IL-18 Tg mouse with Balb/c IL-13 (−/−) mice, as reported previously [18]. Juvenile female WT Balb/c mice, aged 6–7 weeks, were obtained from Kyudo Co., Ltd. (Saga, Japan). All procedures were approved by the Committee on the Ethics of Animal Experiments, Kurume University (Approval No. H22-079-084). Animal care was provided in accordance with the procedures outlined in the “Principle of laboratory animal care” (National Institutes of Health Publication No.86-23, revised 1985).

### Study Design for Mouse Asthma Model

**Figure 1:**
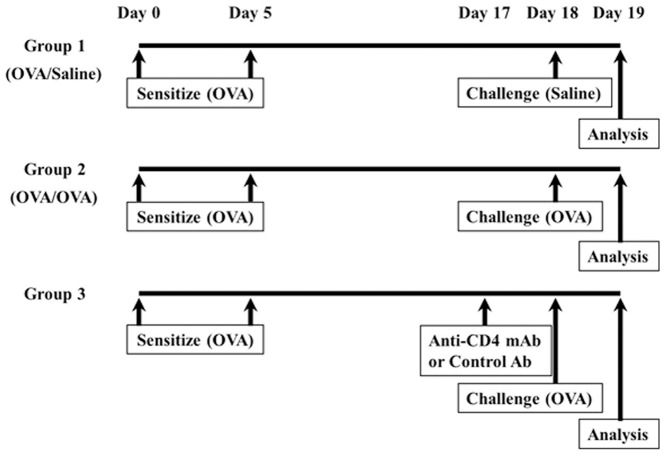
Study design for mouse asthma model. Group 1: ovalbumin (OVA)-sensitized mice injected intraperitoneally with 10 µg of OVA plus 4 mg of Al (OH)3 on day 0 and 5 and saline-challenged on day 18 with 0.9% saline for 20 min; group 2: OVA-sensitized mice injected intraperitoneally with OVA on day 0 and 5 and OVA-challenged on day 18 with 5% OVA (w/v) in 0.9% saline for 20 min; group 3: ovalbumin (OVA)-sensitized mice injected intraperitoneally on day 0 and 5, treated with an intra-peritoneal injection of purified 500 µg of rat anti-mouse CD4 monoclonal antibody (mAb) (GK1.5, rat IgG2b, k) or rat IgG (Sigma–Aldrich Chemical) on day 17 and OVA-challenged on day 18, the airway hyperresponsiveness (AHR) and bronchoalveolar lavage fluid (BALF) were analyzed in all mice.

The experimental protocol, as we previously reported [19], is outlined in Fig. 1. Briefly, mice were divided into three groups. Group 1 mice were treated on day 0 and 5 with an intra-peritoneal injection of 10 µg sterile chicken ovalbumin (OVA, grade V, Sigma–Aldrich Chemical, St. Louis, MO) emulsified with 4 mg of sterile aluminum hydroxide (Alu-Gel-S Suspension, Serva Electrophoresis GmbH, Heidelberg, Germany) in a total volume of 200 µL. On day 18, mice were challenged for 20 min with saline, given via the airways by ultrasonic nebulizer. Groups 2 were sensitized with OVA as described for group 1 and challenged with 5% OVA in 0.9% saline as described for group 1. Groups 3 mice were treated with an intra-peritoneal injection of purified 500 µg of rat anti-mouse CD4 monoclonal antibody (mAb) (GK1.5, rat IgG2b, *k*) or rat IgG (Sigma–Aldrich Chemical) on day 17, as previously reported [8], [20]. On day 18, mice were challenged with OVA as described for group 2. On day 19, we analyzed airway hyperresponsiveness (AHR), bronchoalveolar lavage fluid (BALF) and histological analysis in all groups, as we previously reported [19] [21].

### Histological Examinations

For the histological analysis, mice were sacrificed with an intraperitoneal injection of sodium pentobarbital (2.5–5 mg per mouse). After the thorax had been opened, the lungs were immediately fixed by intratracheal instillation of 10% buffered formalin for 15 to 20 minutes at a constant pressure of 27 cm H_2_O. After gross examination, the extracted tissues were placed into 20% buffered formalin and further fixed for at least 24 hours. Sections (4-µm thick) were cut from paraffin-embedded tissues, placed on poly-l-lysine–coated slides, and then incubated overnight at 55 to 60°C. Deparaffinized sections were stained with hematoxylin and eosin (HE), as reported [19]–[21].

### Isolation of Bronchoalveolar Lavage Fluid (BALF) from Mice

It is possible that over-lavage fluid induces lung injury in mice. Therefore, in this study, we used age-matched female WT and Tg mice to obtain similar body weights of the mice. (i.e. 18 to 20 g). The trachea was inserted with a tubing adaptor, and the lungs were washed three times with 3 mL PBS. Aliquots of cells were centrifuged onto glass slides, dried in air, and stained with Wright-Giemsa. Cell populations were counted, and absolute number of cell populations were then calculated, as previously reported [19]. The remaining BALF was centrifuged, and the supernatants were then collected and stored at –80°C until ELISA assay.

### Surface Ag and Intracellular Analysis by Flow Cytometry

On day 19, BALF cells were isolated from OVA-sensitized and challenged mice. Flow cytometric analysis was performed using a FC500® flow cytometer (Beckman Coulter, Palo Alto, CA) as reported [22]. Anti-mouse CD16/CD32 mAb (2.4G2, PharMingen, San Diego, CA) was used to block the non-specific binding. Isolated BALF cells from mice were stained with PC5-anti-mouse CD4 mAb, FITC-anti-mouse CD8 mAb, and/or control isotype matched mAbs (eBioscience, San Diego, CA). Absolute number in cell populations was then calculated, as previously reported [19].

Three-color analysis was performed for intracellular cytokine staining. Isolated BALF cells were stained with FITC-anti-mouse CD4 mAb, or control FITC-isotype matched mAb. The BALF cells were treated in 1% paraformaldehyde and 0.1% saponin. Then, the BALF cells were further stained with PE-anti-mouse IL-13 mAb, PC7-anti-mouse IFN-γ mAb, and/or control isotype matched mAbs, as reported previously [14], [22]. The lymphocyte population was gated for intracellular cytokine analysis.

### ELISA Assays

The whole lung tissues were homogenized in 2 mL of lysis buffer (1% Triton X-100, 10 mM Tris-HCl, 5 mM EDTA, pH 7.6) containing a protease inhibitor cocktail (Complete™ Mini, Boehringer Mannheim GmbH, Mannheim, Germany) and centrifuged at 20,000×*g* for 15 min as reported [14]. The concentrations of mouse IFN-γ, IL-1β, IL-5, IL-12p70, IL-13, IL-17A/F, and eotaxin in the lungs and BALFs were measured by μµspecific ELISA kits from R&D Systems (Minneapolis, MN).

### Assessment of Airway Hyperresponsiveness

Mice were anesthetized with sodium pentobarbital intraperitoneally, and their tracheas were cannulated via tracheostomy. The mice were ventilated mechanically (tidal volume, 325 µl; frequency, 120 breaths/minute) as reported [21]. A paralytic agent (pancuronium bromide) was administered. Airway opening pressure was measured with a differential pressure transducer and was recorded continuously. Stepwise increases of acetylcholine chloride (ACh, catalog no. A-6625, Sigma–Aldrich Chemical) in 0.9% saline (0.625 to 160 mg/ml) were given by an ultrasonic nebulizer (30 seconds). The data were expressed as the provocative concentration 200 (PC200); the concentration at which airway pressure was 200% of its baseline. PC200 was calculated by log-linear interpolation for individual mice. We also show the results of airway resistance changes from baseline in response to 9 different doses of Ach (0, 0.625, 1.25, 2.5, 5, 10, 20, 40, 80, and 160 mg/ml).

### Measurement of Serum Mouse Total IgE and OVA-specific IgE Levels

Serum Total IgE and OVA-specific IgE concentrations were measured as previously described [19]. Briefly, ELISA plates were coated with 50 µL of 2 µg/mL of two different anti-IgE mAbs (clones R35-72 [PharMingen] and LO-ME-3 [Biosource, Camarillo, CA]) in PBS, washed, and blocked with PBS containing 1% bovine serum albumin. Diluted (1∶10) serum samples were added and incubated overnight at 4°C. Plates were washed and incubated with 3 µg of biotin-labeled OVA in PBS, washed, incubated with streptavidin-horseradish peroxidase conjugate, washed, and developed with ELISA POD substrate TMB kit (Nacalai tesque, Kyoto, Japan). Absorbance was read at 450 nm.

### Statistical Analyses

Results are expressed as means ± standard error of the mean (SEM). ANOVA was used to compare differences between groups. SAS 9.1.3 software, Japanese edition (SAS Institute, Cary, NC, USA), was used for statistical analysis. P<0.05 was considered to represent statistical significance.

## Results

### Establishment of Lung-specific Balb/c Background IL-18 Tg Mice

As we previously reported [16], very severe pulmonary inflammation and emphysema are induced in C57BL/6 lung-specific IL-18 Tg mice and recently, Elias and his colleagues reported similar results [23]. Previous studies have used female BALB/c background mice to examine AHR and pulmonary inflammation in mouse asthma models [19]. Therefore, in this study, we bred the lung-specific IL-18 Tg mice onto a BALB/c genetic background. We established IL-18 Tg mice on the Balb/c genetic background by backcrossing B6 IL-18 Tg mice with WT Balb/c mice 8 times. The genetics are at the 8^th^ generation since mice are typically backcrossed to the 10th generation. Severe pulmonary inflammation and emphysema were observed in the lungs of B6 IL-18 Tg mice as we previously reported [16]. However, pulmonary inflammation and emphysema were not observed in the lungs of IL-18 Tg mice backcrossed with WT Balb/c mice more than 6 times (data not shown). ELISA analysis showed the levels of IL-18 in the whole lungs of 9-week-old female Balb/c IL-18 Tg mice and WT Balb/c mice were 6647.1±1320.5 pg/mL (n = 7) and 299.4±32.0 pg/mL (n = 6), respectively. The levels of IFN-γ in the whole lungs of 9-week-old female Balb/c IL-18 Tg mice and WT Balb/c mice were 184.1±67.4 pg/mL (n = 7) and 90.3±21.7 pg/mL (n = 7), respectively. The levels of IL-13 in the whole lungs of 9-week-old female Balb/c IL-18 Tg mice and WT Balb/c mice were 3.0±0.7 pg/mL (n = 7) and 3.8±1.6 pg/mL (n = 6), respectively. The level of IL-18 and IFN-γ but not IL-13 protein were significantly (p<0.05) increased in the lungs of IL-18 Tg mice as compared to the lungs of WT mice.

### Severe Airway Inflammation in the IL-18 Tg Mice Mouse Asthma Model

**Figure 2:**
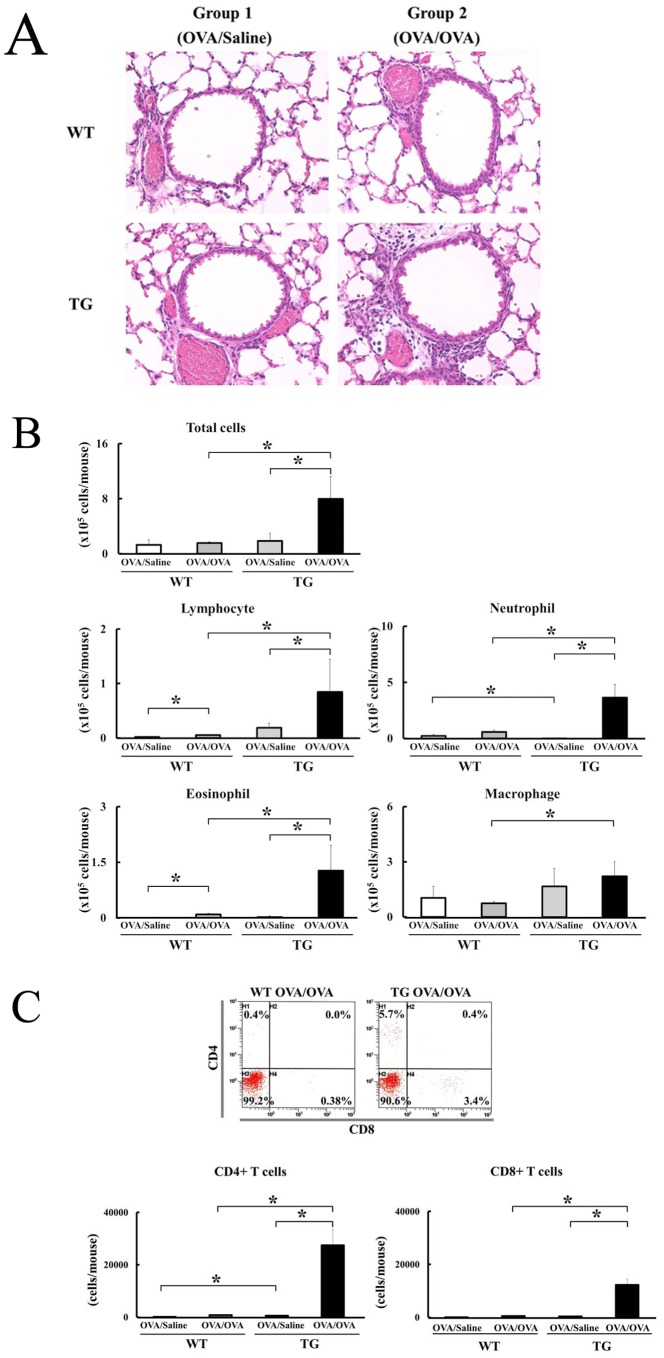
Severe airway inflammation accompanied with CD4^+^ T cells and eosinophils in IL-18 Tg mice in a mouse asthma model. (A) Histological evidence of airway inflammation in OVA-sensitized and challenged mice (group 1). Lung tissues were also obtained from OVA-sensitized and saline-challenged mice (group 2). Original magnification 400X, HE staining. (B) Cells in the BALFs were centrifuged onto glass slides, dried in air, and stained with Wright–Giemsa. Cell populations in the BALFs were calculated as described under Materials and methods. (n = 4 to 6 per each group) *: p<0.05 (C) Flow cytometric analysis was performed to examine CD4^+^ T cells and CD8^+^ T cells using a FC500® flow cytometer (Beckman Coulter, Palo Alto, CA). Anti-mouse CD16/CD32 mAb (2.4G2, PharMingen, San Diego, CA) was used to block the non-specific binding. Isolated BALF cells from mice were stained with PC5-anti-mouse CD4 mAb, FITC-anti-mouse CD8 mAb, and/or control isotype matched mAbs (eBioscience, San Diego, CA). Cell populations were calculated as described under Materials and methods. (n = 4 to 6 per each group) *: p<0.05.

In this study, we used a conventional mouse asthma model for antigen (OVA)-sensitization and airway challenged mice [19]. Histological examination by HE staining showed severe airway inflammation in the lungs of OVA–sensitized and OVA–challenged (OVA/OVA–) IL-18 Tg mice, as compared to OVA/OVA–WT mice (group 2 in Fig. 2A). In contrast, airway inflammation was not observed in the lungs of OVA–sensitized and saline–challenged (OVA/saline–) IL-18 Tg mice and WT mice (group 1 in Fig. 2A). To confirm this observation, we have analyzed the cell population of the BALFs in groups 1 and 2 on day 20. Eosinophils and lymphocytes were significantly increased in the BALFs of OVA/OVA–WT mice as compared to OVA/saline–WT mice. Total cell number, lymphocytes, neutrophils, and eosinophils were significantly increased in BALFs of OVA/OVA–IL-18 Tg mice, compared with OVA/saline–IL-18 Tg mice (Fig. 2B). Next, we analyzed the increasing lymphocytes by flow cytometry (Fig. 2C). The absolute number of CD4^+^ and CD8^+^ T cells was significantly increased in the BALFs of OVA/OVA–IL-18 Tg mice, as compared to OVA/OVA–WT mice and OVA/saline–IL-18 Tg mice.

### Overproduction of IFN-γ and IL-13 in OVA-sensitized and Challenged IL-18 Tg Mice

**Figure 3:**
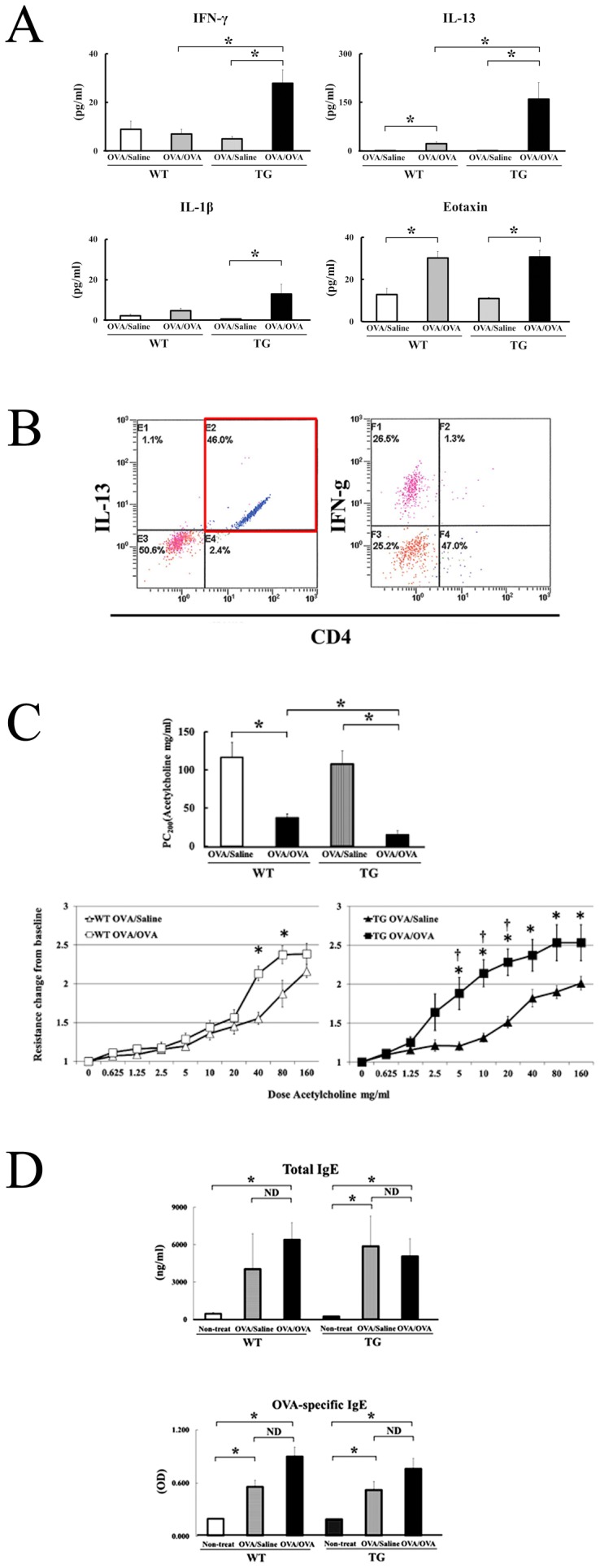
Overproduction of IL-18 in lungs increases Th1 and Th2 cytokines, and airway hyperresponsiveness. (A) The concentrations of IFN-γ, IL-13, IL-1β, and eotaxin in the bronchoalveolar lavage fluids (BALFs) were measured by specific ELISA kits. (n = 4 to 6 per each group) *: p<0.05 (B) On day 19, BALF cells were isolated from OVA-sensitized and challenged mice. Three-color analysis was performed for intracellular cytokine staining. Isolated BALF cells were stained with FITC-anti-mouse CD4 mAb, PE-anti-mouse IL-13 mAb, PC7-anti-mouse IFN-γ mAb, and/or control isotype matched mAbs, as reported previously [14]. The lymphocyte population was gated for intracellular cytokine analysis. (C) Mice were anesthetized intraperitoneally with sodium pentobarbital, and their tracheas were cannulated via tracheostomy. The mice were ventilated mechanically (tidal volume, 325 µl; frequency, 120 breaths/minute). A paralytic agent (pancuronium bromide) was administered and airway opening pressure was measured with a differential pressure transducer and was recorded continuously. Stepwise increases of acetylcholine chloride (ACh, catalog no. A-6625, Sigma–Aldrich Chemical) in 0.9% saline (0.6 to 160 mg/ml) were given by an ultrasonic nebulizer (30 seconds). The data were expressed as the provocative concentration 200 (PC200); the concentration at which airway pressure was 200% of its baseline. PC200 was calculated by log-linear interpolation for individual mice. The data were also expressed as airway resistance changes from baseline in response to 9 different doses of Ach (0, 0.625, 1.25, 2.5, 5, 10, 20, 40, 80, and 160 mg/ml). (n = 6 to 10 per each group) *: p<0.05. (D) Serum Total IgE and OVA-specific IgE concentrations were measured. (n = 5 to 12 per each group) *: p<0.05.

We analyzed protein levels of IFN-γ, IL-1β, IL-5, IL-12p70, IL-13, IL-17A/F, and eotaxin in the BALFs of groups 1 and 2. The concentrations of IFN-γ and IL-13 were significantly increased in the BALFs of OVA/OVA–IL-18 Tg mice, as compared to OVA/OVA–WT mice. In contrast, there was no significant difference in the concentrations of IL-5, IL-12p70, and eotaxin between OVA/OVA–IL-18 Tg and OVA/OVA–WT mice. Moreover, IL-17A/F was under detectable level in the BALFs of OVA/OVA–IL-18 Tg and WT mice (Fig. 3A and data not shown).

### IL-13 Producing CD4^+^ T Cells were Increased in Lungs of OVA-sensitized and Challenged IL-18 Tg Mice

We performed the intercellular staining to examine whether IL-18 induces IL-13 and/or IFN-γ in CD4^+^ T cells. We obtained BALF cells from OVA-sensitized and challenged IL-18 Tg miceand intracellular staining analysis revealed that overproduction of IL-18 protein in the lungs induces IL-13 but not IFN-γ in CD4^+^ T cells (Fig. 3B).

### Overproduction of IL-18 in Lungs Increases Airway Hyperresponsiveness but not IgE

We investigated airway hyperresponsiveness (AHR) in OVA/saline–IL-18 Tg and WT mice on day 19 (Fig. 3C). Interestingly, AHR was not increased in OVA/saline–IL-18 Tg mice or OVA/saline–WT mice. In contrast, AHR in OVA/OVA–IL-18 Tg mice was significantly increased as compared to OVA/OVA–WT mice.

As reported previously, the level of OVA-specific IgE increases in the sera of mouse asthma model using OVA-sensitized and challenged Balb/c mice [19]. Therefore, we examined whether the levels of total IgE and/or OVA-specific IgE are increased in the sera of OVA/OVA–IL-18 Tg mice. While the levels of total IgE and OVA-specific IgE were increased in the sera of OVA/OVA–WT and Tg mice, there was no significant difference of the level of total IgE and OVA-specific IgE when comparing OVA/OVA–WT and Tg mice (Fig. 3D). These results suggest that AHR was not increased in naïve IL-18 Tg mice, and OVA-specific IgE may not directly induce AHR in OVA/OVA–IL-18 Tg mice.

### Administration of Anti-CD4 mAb Improves AHR in the IL-18 Tg Mice

**Figure 4:**
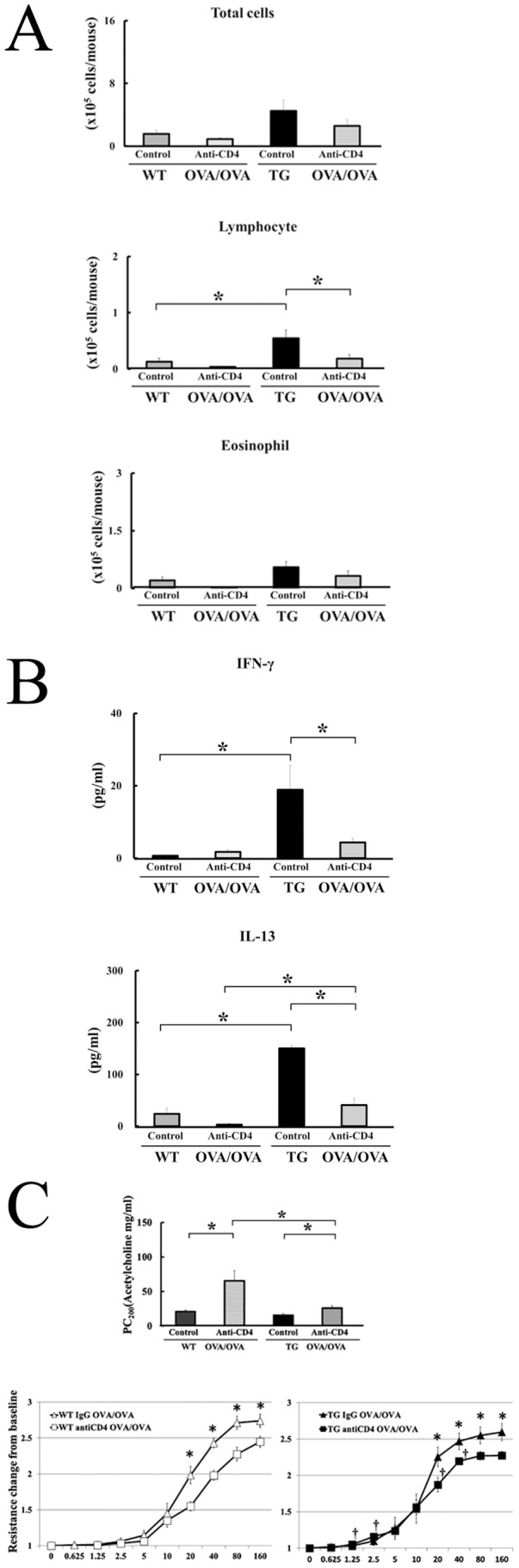
Treatment with anti-CD4 mAb decreased airway inflammation, Th1 and Th2 cytokines, and airway hyperresponsiveness in IL-18 Tg mice. In group 3, OVA-sensitized mice were treated with 500 µg of rat anti-mouse CD4 mAb or rat IgG on day 17, and OVA-challenged on day 18. (A) Cell populations in the BALFs. (n = 5 to 7 per each group) *: p<0.05. (B) The concentrations of mouse IFN-γ and IL-13 in the BALFs were measured. (n = 5 to 7 per each group) *: p<0.05. (C) Airway responsiveness to aerosolized acetylcholine was examined as described above. The data were also expressed as airway resistance changes from baseline in response to 9 different doses of Ach (0, 0.625, 1.25, 2.5, 5, 10, 20, 40, 80, and 160 mg/ml). (n = 7 to 10 per each group) *: p<0.05.

In these studies, we found that CD4^+^ and CD8^+^ T cells were significantly increased in the BALFs of OVA/OVA–IL-18 Tg mice, as compared to control WT mice. Of note, the number of CD4^+^ T cells was greater than CD8^+^ T cells in the BALFs of OVA/OVA–IL-18 Tg mice (Fig. 2C). Moreover, the intracellular staining analysis revealed overproduction of IL-18 protein in the lungs increased IL-13 producing CD4^+^ T cells (Fig. 3B). Therefore, we investigated whether deletion of CD4^+^ T cells results in decreasing AHR in OVA/OVA–IL-18 Tg mice. We used anti-mouse CD4 mAb (GK1.5) or control Ab (rat IgG) on day 18 (24 hours before challenge with OVA) to deplete CD4^+^ T cells *in vivo*. On day 19, BALF, levels of cytokines and AHR were analyzed (group 3 in Fig. 1). Treatment with anti-CD4 mAb significantly decreased lymphocytes, but not total cells, eosinophils, neutrophils, or alveolar macrophages in the in the BALFs of OVA/OVA–IL-18 Tg mice as compared to control Ab (Fig. 4A). Treatment with anti-CD4 mAb also significantly decreased the levels of IL-13 and IFN-γ in the BALFs of OVA/OVA–IL-18 Tg mice as compared to control Ab (Fig. 4B). Moreover, anti-CD4 mAb significantly decreased AHR in OVA/OVA–IL-18 Tg mice as compared to control Ab (Fig. 4C).

### Deletion of IL-13 Gene Improves AHR in IL-18 Tg Mice

**Figure 5:**
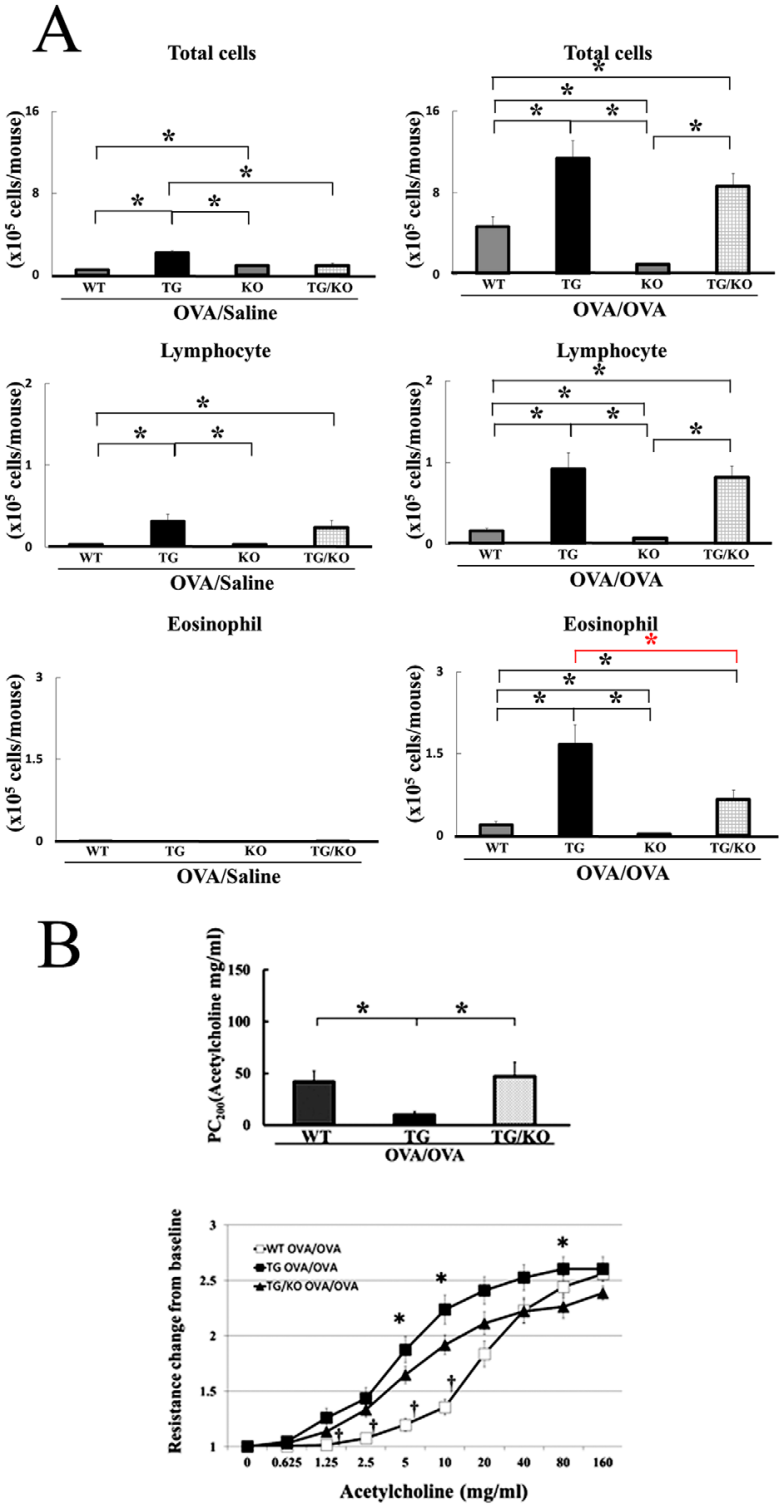
Deletion of IL-13 gene decreased airway inflammation, Th1 and Th2 cytokines, and airway hyperresponsiveness in IL-18 Tg mice. WT Balb/c, IL-18 Tg, IL-13 gene deficient (KO), and IL-18 Tg/IL-13 gene deficient (TG/KO) mice were OVA-sensitized and challenged with OVA or saline. (A) Cell populations in the BALFs were calculated. (n = 14 to 20 per each group) *: p<0.05. (B) Airway responsiveness to aerosolized acetylcholine was examined as described above. The data were also expressed as airway resistance changes from baseline in response to 9 different doses of Ach (0, 0.625, 1.25, 2.5, 5, 10, 20, 40, 80, and 160 mg/ml). (n = 14 to 22 per each group) *: p<0.05.

Next we focused on determining the effect of the increased levels of IL-13 in the lungs of OVA/OVA–IL-18 Tg mice. Thus we established IL-18 Tg mice lacking the IL-13 gene (IL-18 Tg/IL-13KO mice) by backcrossing with Balb/c IL-13 deficient mice. As shown in Fig. 2B, the total cell number, lymphocytes, eosinophils, neutrophils, and macrophages were significantly increased in the BALF of OVA/OVA–IL-18 Tg mice as compared to OVA/OVA–WT mice. Interestingly, the number of eosinophils (but not the total cell number, lymphocytes, neutrophils, nor macrophages) in the in the BALFs of OVA/OVA–IL-18 Tg/IL-13KO mice were significantly decreased as compared to the BALFs of OVA/OVA–IL-18 Tg mice (Fig. 5A). Moreover, AHR in the OVA/OVA–IL-18 Tg/IL-13KO mice was significantly decreased as compared to OVA/OVA– IL-18 Tg mice (Fig. 5B).

## Discussion

It has been suggested that IL-18 may play an important role in the pathophysiology of patients with asthma. Higher serum levels of IL-18 have been previously identified in asthmatic subjects in comparison to healthy control subjects [24]. In addition, significantly higher serum IL-18 levels have been reported in patients with acute severe asthma [25]. An IL-18 gene polymorphism was reported to be associated with asthma severity; the rs5744247 variant reflecting both higher transcriptional activity and higher serum IL-18 levels [26]. In addition, the IL-18R gene (on 2q21) has been identified as a candidate gene associated with increased susceptibility to asthma in children [27], and polymorphisms of the gene are related to allergic asthma and airway hyper-responsiveness (AHR) [28]. We found that the IL-18 protein was strongly expressed in airway epithelium cells and smooth muscle cells in airway biopsy samples from allergic asthmatic subjects. Moreover, serum levels of IL-18 were significantly higher in the asthmatic subjects than in either the non-asthmatic allergic subjects or the healthy controls [29]. In contrast, IL-18Rα was weakly expressed in the airway epithelium, and not on airway smooth muscle cells. In this study, we showed that AHR and airway inflammatory cells accompanied with CD4^+^ T cells, CD8^+^ T cells and eosinophils were significantly increased in IL-18 Tg mice sensitized– and challenged– with OVA (OVA/OVA–IL-18 Tg mice), as compared to control WT mice. Treatment with anti-CD4 mAb significantly decreased the number of lymphocytes and AHR in the lungs of OVA/OVA–IL-18 Tg mice, as compared to control Ab treated OVA/OVA–IL-18 Tg mice. The intracellular staining analysis revealed that overproduction of IL-18 protein in the lungs induces IL-13 but not IFN-γ in CD4^+^ T cells. We utilized anti-CD4 mAb and IL-13 gene deficient (−/−) mice, and shown reduced airway inflammation and AHR following OVA-challenge in the IL-18 Tg mice. Our data shows that overproduction of IL-18 protein in the lungs increases pulmonary inflammation accompanied with IL-13 producing CD4^+^ T cells, and results in increasing AHR in this mouse asthma model.

Previously, we showed that constitutive overproduction of mature IL-18 protein in the lungs of B6 background transgenic mice resulted in the increased production of IFN-γ IL-5, and IL-13**,** and severe emphysema accompanied by pulmonary inflammation, especially by CD8^+^ T cells. Moreover, disruption of the IL-13 but not IFN-γ gene prevented emphysema and pulmonary inflammation in IL-18 Tg mice [16]. A recent study by Kang and colleagues demonstrated that the inducible expression of IL-18 in the mature murine lung induces pulmonary inflammation with the accumulation of CD4^+^, CD8^+^, CD19^+^ and NK1.1^+^ cells, emphysema, mucus metaplasia, airway fibrosis, vascular remodeling and right ventricle cardiac hypertrophy in B6 mice. Moreover, disruption of the IL-13, IL-17 gene, but not the IFN-γ gene prevented emphysema and pulmonary inflammation in IL-18 Tg mice [23]. We previously reported that *in vivo* overexpression of IL-18 alone is not sufficient to elicit lung disease, since lymphocyte-specific IL-18 B6 Tg mice [9], and skin-specific IL-18 B6 Tg mice [10] did not exhibit pulmonary inflammation or emphysema. Additionally, conditional lung-specific IL-13 expression with IFN-γ overproduction in the lungs induces emphysema in adult mice [30]. In this study, we established lung-specific IL-18 Tg mice Balb/c background by backcrossing the B6 lung-specific IL-18 Tg mice with WT Balb/c mice. Expression levels of IL-18 and IFN-γ but not IL-13 were increased in the lungs of Balb/c IL-18 Tg mice. Histological analysis found that the lung tissues of Balb/c IL-18 Tg mice were quite normal. Thus, we believe that localized production of IL-18 in the lungs may play an important role in the development of pulmonary inflammation and emphysema via IL-13 production in mice as well as COPD patients. Further studies will be needed to elucidate the molecular mechanisms involved with the lack of IL-13 gene expression in the lungs of Balb/c IL-18 Tg mice.

IL-18 was reported to take part in the differentiation of Th17 cells by amplifying IL-17 production by polarized Th17 cells in synergy with IL-23 [31], [32]. As described above, the inducible expression of IL-18 in the lungs induces pulmonary inflammation, emphysema, mucus metaplasia, airway fibrosis, vascular remodeling and right ventricle cardiac hypertrophy in adult B6 mice using the CC10 promoter. In addition, disruption of the IL-17 gene prevented emphysema and pulmonary inflammation in the B6 IL-18 Tg mice [23]. In this study, there was no significant difference in the expression levels of IL-5, IL-12p70, and eotaxin between OVA/OVA–IL-18 Tg and OVA/OVA–WT mice. Moreover, IL-17A/F was under detectable level in the BALFs of OVA/OVA–IL-18 Tg and WT mice, suggesting that Th17 cells may not play a role in our mouse asthma model. However, there are several studies that have demonstrated the important effects of IL-17 in the pathogenesis of asthma [33]–[35]. It has been shown that many of these cytokine responses are very transient, so. it is possible that the response was earlier or later and that the transgenic mouse response kinetics may not be the same as the wild type mouse. Further analysis will be needed to address this issue.

Currently, therapeutic approaches for steroid-resistant uncontrolled asthma are limited [36]. Our present results suggest that overexpression of IL-18 in the lungs may induced pulmonary inflammation and AHR, and result in exacerbating the disease activities in patients with asthma. Blocking of IL-18 expression may be feasible *in vivo*. IL-18 inhibitors, including caspase-1 inhibitors, anti-human IL-18 monoclonal antibodies, anti-human IL-18R monoclonal antibodies, soluble IL-18 receptor complex [11] and/or IL-18 binding protein (BP) [37] may be clinically beneficial for the treatment of patients with steroid-resistant asthma, for whom current treatment options are very limited.
